# The Association Between Gastroesophageal Reflux Disease and Atrial Fibrillation: A Systematic Review and Meta-Analysis

**DOI:** 10.7759/cureus.78356

**Published:** 2025-02-01

**Authors:** Tanya Sinha, Heer M Joshi, Bansari Patel, Hashmatullah Stanikzai, Helai Hussaini, Sandipkumar S Chaudhari, Ihtisham Habib, Shamsha Hirani

**Affiliations:** 1 Internal Medicine, Tribhuvan University, Kathmandu , NPL; 2 Internal Medicine, Jackson Park Hospital, Chicago, USA; 3 School of Medicine, American University of Barbados, Bridgetown, BRB; 4 Medicine, Shaikh Khalifa Bin Zayed Al-Nahyan Medical and Dental College, Lahore, PAK; 5 Ear, Nose, and Throat, West Anaheim Medical Centre, Anaheim, USA; 6 Cardiothoracic Surgery, University of Alabama at Birmingham, Birmingham, USA; 7 Family Medicine, University of North Dakota School of Medicine and Health Sciences, Fargo, USA; 8 Internal Medicine, Medical Teaching Institute, Lady Reading Hospital, Peshawar, PAK; 9 Cardiology, Baqai Hospital, Karachi, PAK

**Keywords:** atrial fibrillation, cardiovascular disease, gastroesophageal reflux disease, inflammation, meta-analysis

## Abstract

The relationship between gastroesophageal reflux disease (GERD) and atrial fibrillation (AF) has been increasingly recognized, but its nature and strength remain unclear. We conducted a systematic review and meta-analysis of studies from January 2010 to November 2024 using PubMed, Excerpta Medica Database (EMBASE), and Web of Science databases. Seven studies were included: three cohort studies, two Mendelian randomization studies, one case-control study, and one cross-sectional study. Meta-analysis revealed that GERD was associated with a significantly increased risk of AF (RR: 1.27, 95% CI: 1.15-1.40). This association remained robust in sensitivity analyses. The two Mendelian randomization studies provided genetic evidence supporting a potential causal relationship. The proposed mechanism involves inflammatory pathways extending from the esophagus to the left atrium. The analysis was constrained by the small number of studies, methodological heterogeneity (I-Square: 81%), and limited ability to perform subgroup analyses. The findings suggest that GERD patients may benefit from AF screening, and GERD management could potentially modify AF risk. Future research should focus on prospective studies examining the impact of GERD treatment on AF prevention and progression, as well as identifying high-risk subgroups who might benefit most from targeted interventions.

## Introduction and background

Atrial fibrillation (AF) stands out as the most frequently observed arrhythmia in clinical cardiology, affecting 1% to 2% of the general population. Its prevalence is expected to double or even triple over the next 20 to 30 years [1,2], driven by an aging population, evolving lifestyle patterns, and suboptimal management of cardiovascular risk factors [3]. The clinical significance of AF lies in its substantial impact on quality of life and its association with serious complications, including heart failure and cardiogenic stroke [4].

While the traditional risk factors for AF are well-documented, emerging evidence suggests that gastroesophageal reflux disease (GERD) may play an important role in its development and progression. GERD affects approximately 20% of the United States population and can manifest across all adult age groups [5]. The condition extends beyond simple acid reflux, presenting as a complex, multifactorial disorder often associated with sliding hiatus hernia and various lifestyle factors, particularly in middle-aged adults (40-60 years). These lifestyle factors include sleep apnea, obesity, smoking, and excessive alcohol consumption [6,7].

Recent research has begun to illuminate the potential mechanistic links between GERD and cardiovascular conditions, particularly AF [8]. The anatomical proximity of the esophagus to the left atrium creates a pathway for local inflammation to affect cardiac function. This inflammatory process, combined with shared risk factors such as obesity and sleep apnea, may explain the observed association between these conditions [9,10]. The inflammatory cascade initiated by GERD could potentially trigger or maintain atrial arrhythmias, as suggested by studies showing improvement in cardiac rhythm with acid-suppressive therapy [11].

The relationship between GERD and AF has gained increasing attention in recent years. A meta-analysis by Xu et al. provided initial evidence of this association [12]. However, since its publication, numerous observational studies and clinical investigations have emerged, offering new insights into the strength and consistency of this relationship. These newer studies explore potential mechanisms linking the two conditions and examine variations across different patient subgroups.

Despite these advances, several questions remain unanswered regarding the precise nature and clinical implications of the GERD-AF relationship. Understanding this association is crucial for several reasons: it could identify GERD as an independent risk factor for AF, inform screening strategies for early AF detection in GERD patients, and potentially guide therapeutic approaches to mitigate AF risk through GERD management. Therefore, an updated systematic review and meta-analysis is warranted to synthesize the accumulating evidence and provide a comprehensive, current understanding of the relationship between GERD and AF. This knowledge would be invaluable for clinicians, researchers, and policymakers in developing evidence-based strategies for patient care.

The primary aim of this systematic review and meta-analysis is to assess the relationship between GERD and AF, incorporating recent evidence to better understand their association and its clinical implications.

## Review

Methodology 

Search Strategies 

A systematic search was performed using online databases such as PubMed, Excerpta Medica Database (EMBASE), and Web of Science from January 2010 to November 25, 2024. The following keywords were used: “gastroesophageal reflux,” “gastroesophageal reflux disease,” “GERD,” “esophageal reflux,” AND “atrial fibrillation,” “auricular fibrillation,” and “AF.” Boolean algebra operators were used along with medical subject heading (MeSH) terms to further optimize the search. The meta-analysis of observational studies in epidemiology (MOOSE) guidelines was applied. Additionally, a reference list of all included articles was manually screened to find additional relevant articles. The search was performed by two authors independently. Disagreements were resolved through discussion. 

Eligibility Criteria 

We included all studies that met the following eligibility criteria: original studies, including observational (case-control, cross-sectional, or cohort) and randomized controlled trials (RCTs); studies that expressed the association between AF and GERD. We excluded reviews, editorials, and non-human studies. We excluded studies published in languages other than English. 

Study Selection 

To categorize all possible qualifying studies, two independent researchers looked through the abstracts or titles of the papers that were retrieved from various electronic resources. Following the acquisition of potentially pertinent research, the complete articles were evaluated for conformance with the requirements for inclusion. After reviewing the original data again and consulting with a third reviewer, any confusion or discrepancy between the two investigators was resolved by consensus. 

Data Extraction 

Two authors reviewed and extracted the relevant data from each of the included studies. Relevant data obtained from included studies were the author's name, year of publication, study design, region where the study was conducted, number of patients with GERD, number of patients who developed AF, adjusted odds ratio (OR), risk ratio (RR), and hazard ratio (HR) with 95% confidence interval and basic participant characteristics. 

Statistical Analysis 

We evaluated the association between GERD (or AF) and the risk of developing AF (or GERD) by utilizing relative risks (RRs), odds ratios (ORs), and 95% confidence intervals (CIs) as reported in the included cohort and case-control studies. Odds ratios and incidence rate ratios were interpreted as equivalent to RRs. When necessary, 95% of CIs were derived from the p-values provided in the respective studies. Study heterogeneity was assessed using both Cochrane’s Q test and the I² statistic. For the Q test, a p-value < 0.1 indicated significant heterogeneity, while for the I² statistic, heterogeneity was classified as follows: <30% (minimal heterogeneity), 30-75% (moderate heterogeneity), and >75% (substantial heterogeneity). Combined RRs were calculated using fixed-effects models unless significant heterogeneity was present, in which case random-effects models were employed. Forest plots were created to visually represent the RRs and their 95% CIs for individual studies and the overall analysis. The cut-off of the p-value was kept at 0.05. All analysis was performed using Review Manager Version 5.4.1 (The Cochrane Collaboration, London, UK). 

Results 

**Figure 1:**
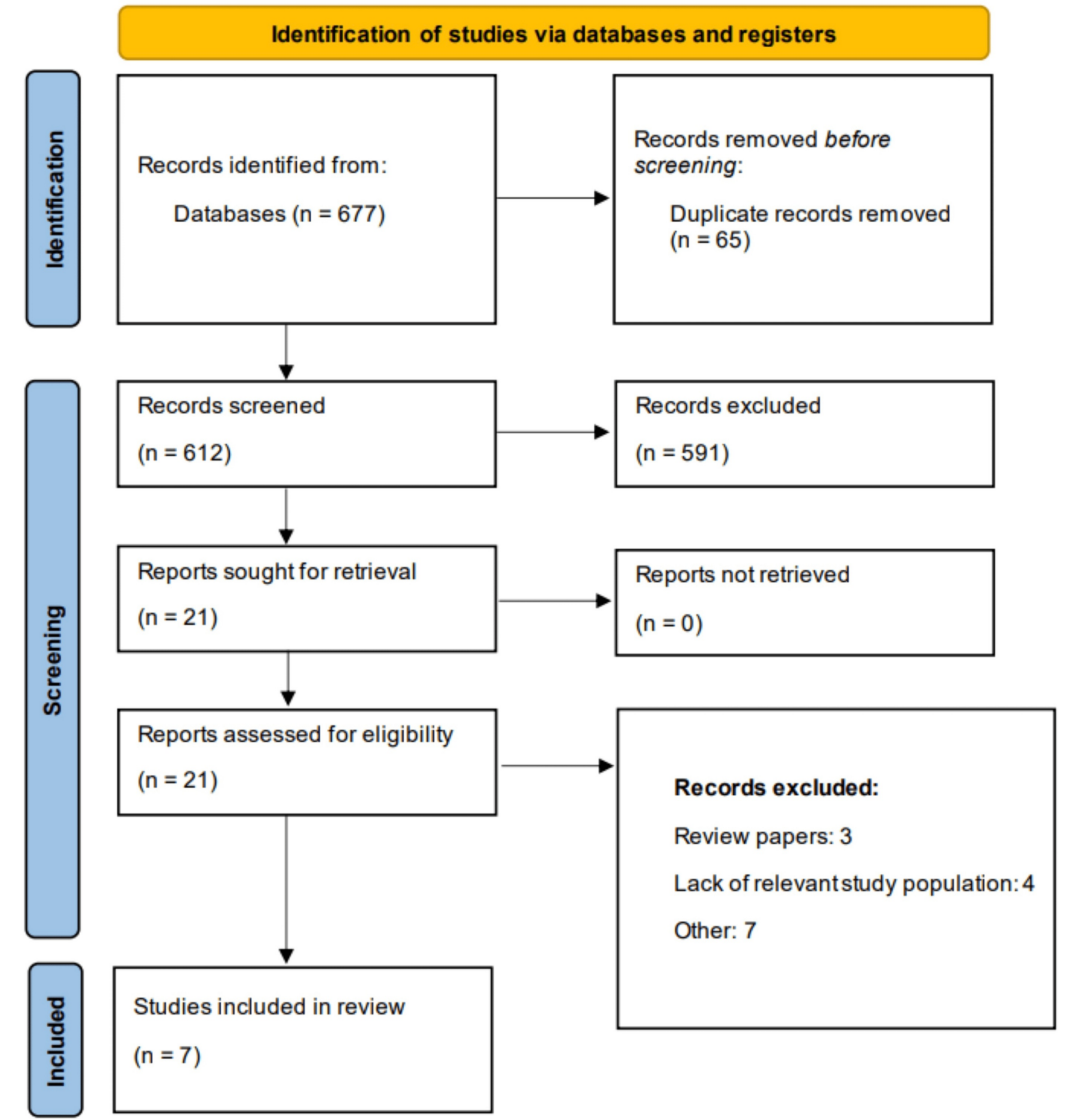
Study selection process (PRISMA flowchart) PRISMA: Preferred Reporting Items for Systematic Reviews and Meta-Analyses

Through online screening, we found 677 potentially relevant studies. After removing duplicates, 612 studies were initially screened using titles and abstracts. After initial screening, 21 studies were found eligible for detailed screening. Finally, seven studies were included in this meta-analysis. Figure 1 presents the Preferred Reporting Items for Systematic Reviews and Meta-Analyses (PRISMA) flowchart of study selection. Table 1 presents the characteristics of the included studies. Overall, three studies were cohort, two were Mendelian randomization studies, one was case-control, and one study was cross-sectional. Two studies were conducted in China, one each in Taiwan, Japan, Romania, Sweden, and the United States. The included studies were published between 2011 and 2024. Table 2 presents the quality assessment of the included studies.

**Table 1:** Characteristics of included studies (n=7) NR: not reported; GERD: gastroesophageal reflux disease; AF: atrial fibrillation

Author	Year	Study design	Region	Number of patients with GERD	Number of patients who developed AF	Age (Years)	Male (n)
Chen et al. [13]	2024	Mendelian randomization study	China	NR	NR	NR	NR
Floria et al. [14]	2017	Case-control	Romania	61	20	NR	25
Huang et al. [15]	2012	Cohort	Taiwan	29668	184	50.99	14373
Maret-ouda et al. [16]	2022	Cohort	Sweden	118013	7042	NR	NR
Shimazu et al. [17]	2011	Cross-sectional	Japan	16	14	70.31	8
Wang et al. [18]	2024	Cohort	United States	2,051,965	362,839	75.1	169110
Wang et al. [19]	2024	Mendelian randomization study	China	NR	NR	NR	NR

**Table 2:** Quality assessment of included studies

Author ID	Selection	Comparison	Assessment	Total
Chen et al., 2024 [13]	4	2	3	Good
Floria et al., 2017 [14]	3	2	2	Good
Huang et al., 2012 [15]	4	2	2	Good
Maret-ouda et al., 2022 [16]	2	1	3	Fair
Shimazu et al, 2011 [17]	4	2	2	Good
Wang et al., 2024 [18]	3	2	2	Good
Wang et al., 202419]	4	1	3	Good

GERD and Risk of Atrial Fibrillation

**Figure 2:**
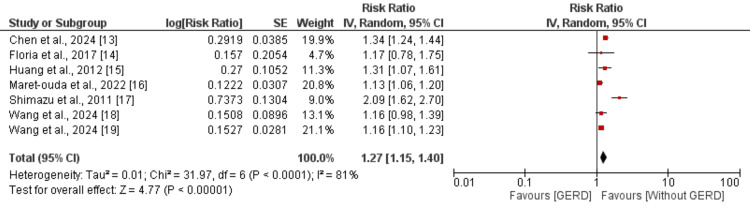
Association of GERD and development of atrial fibrillation GERD: gastroesophageal reflux disorder [13-19]

Seven studies were in the pooled analysis to determine the effect of GERD on the risk of AF, and the results of the pooled analysis are shown in Figure 2. The pooled analysis showed that the risk of AF was significantly greater in patients with GERD compared to the control group (RR: 1.27, 95% CI: 1.15 to 1.40). High heterogeneity is reported among the study results (I-Square: 81%).

Sensitivity analysis was conducted to evaluate the stability of the pooled analysis linking GERD to an increased risk of AF, and the results are presented in Table 3. Excluding each study individually revealed consistent relative risks (RRs), ranging from 1.21 to 1.32, confirming the robustness of the overall estimate (pooled RR: 1.27, 95% CI: 1.15-1.40). Despite these consistent findings, substantial heterogeneity remained across studies (I² = 81%), with individual study heterogeneity ranging from 48% to 84%. The lowest heterogeneity was reported after removing the study performed by Shimazu et al., potentially due to the cross-sectional study design [17]. 

**Table 3:** Sensitivity analysis findings (removing one study at a time) RR: risk ratio; CI: confidence interval

Author	RR (95% CI)	I-Square
Chen et al., 2024 [13]	1.25 (1.12 to 1.40)	78%
Floria et al., 2017 [14]	1.28 (1.15 to 1.42)	84%
Huang et al., 2012 [15]	1.24 (1.14 to 1.41)	84%
Maret-ouda et al., 2022 [16]	1.32 (1.16 to 1.49)	81%
Shimazu et al, 2011 [17]	1.21 (1.12 to 1.29)	48%
Wang et al., 2024 [18]	1.29 (1.16 to 1.44)	84%
Wang et al., 2024 [19]	1.31 (1.15 to 1.51)	83%

Discussion 

This meta-analysis aimed to evaluate the impact of GERD on the risk of developing AF. It included seven studies published between 2011 and 2024, revealing that individuals with GERD have a higher likelihood of developing AF compared to controls. A retrospective, small-scale population study suggested that GERD might increase AF incidence by approximately 39% [20]. Similarly, data from the Taiwan National Health Insurance database indicated an elevated AF risk in GERD patients within a nationwide prospective cohort (HR 1.31; 95% CI: 1.06-16.1) [15]. However, some studies reported inconsistent findings [14,21]. A Mendelian randomization study demonstrated a link between GERD and a higher AF incidence, highlighting the potential benefits of early GERD treatment in reducing AF risk [19]. Despite these findings, the pooled analysis of seven studies showed a non-significant difference in AF risk between GERD patients and controls (RR: 1.06; 95% CI: 0.86-1.3) [12].

Traditional risk factors for AF, such as hypertension, valvular disease, cardiomyopathy, pulmonary conditions, and thyroid disorders, are commonly compared to emerging risk factors like metabolic syndrome and its components, which are often associated with persistent low-grade inflammation. GERD, caused by chemical inflammation from esophageal acid exposure, may contribute to AF through localized inflammation. This inflammation can extend into the left atrium via thin tissue layers, leading to atrial myocarditis or local pericarditis. Acid reflux may also trigger the release of inflammatory mediators like interleukin (IL)-1β and IL-6 [22], which are implicated in AF development. Chronic inflammation, reflected by elevated C-reactive protein (CRP), has been linked to the incidence [23], defibrillation success [24], recurrence [25], and prognosis of AF [26]. GERD and AF may thus be interconnected conditions, sharing inflammatory pathways that sustain and exacerbate both diseases.

Through common anatomical and physiological connections, such as the vagus nerve and pericardial structures, the localized esophageal inflammation brought on by GERD may spread to the left atrium [27]. GERD patients may be more susceptible to AF due to autonomic dysfunction, which is defined by an imbalance in the sympathetic and parasympathetic nervous systems, in addition to inflammatory mediators [28]. It is well recognized that GERD raises vagal tone, which may interfere with normal atrial electrophysiology and cause arrhythmogenesis and ectopic atrial activity [29]. Furthermore, GERD-induced hypoxia brought on by microaspirations or nocturnal acid reflux may exacerbate atrial remodeling and electrical instability by causing oxidative stress and systemic inflammation. Proton pump inhibitors (PPIs) and other acid-suppressive medications may reduce oxidative stress and inflammation, but their potential to prevent AF is still unknown [4].

The causal link between GERD and AF remains uncertain due to potential biases in the observational studies included in this meta-analysis, which may arise from various confounding factors. Establishing causality using traditional epidemiological approaches is challenging. Notably, two of the included studies employed two-sample Mendelian randomization [13,19], a method leveraging genetic variations unrelated to the exposure of interest to mitigate confounding, unlike conventional epidemiological studies. These findings were robust across sensitivity analyses assessing the impact of pleiotropy on causality estimates. Currently, there is insufficient evidence to recommend anti-GERD treatment strategies specifically for AF patients [11]. Further validation of these findings requires advanced research methodologies, including large-scale interventional trials and prospective cohort studies. 

The observed 27% increased risk of AF among GERD patients (RR: 1.27, 95% CI: 1.15-1.40) warrants careful consideration in clinical practice. To contextualize this finding, given that the baseline risk of AF in the general population is approximately 1-2%, a 27% relative increase would translate to an absolute risk increase of 0.27-0.54 percentage points, meaning GERD patients might have a 1.27-2.54% risk of developing AF. While this increase may appear modest for individual patients, its public health impact is substantial considering GERD's high prevalence (affecting 20% of the US population) [1]. Furthermore, this association becomes particularly relevant for GERD patients with other AF risk factors, as the combination could lead to a clinically meaningful cumulative risk. Therefore, clinicians should consider heightened vigilance for AF symptoms in GERD patients, particularly those with additional risk factors such as advanced age, hypertension, or obesity [6]. This may include more frequent cardiac monitoring or earlier diagnostic workups when symptoms suggestive of AF arise in GERD patients.

Future research should aim to clarify the complex relationship between GERD and AF by addressing existing limitations and employing advanced methodologies. Randomized controlled trials (RCTs) are essential to evaluate the impact of GERD management strategies, such as proton pump inhibitors, anti-inflammatory therapies, and lifestyle modifications, on AF incidence and recurrence. Prospective cohort studies with standardized definitions of GERD and AF, along with comprehensive adjustments for confounding factors like comorbidities and lifestyle variables, are needed to provide more robust evidence. Mechanistic studies exploring the molecular and electrophysiological links between GERD and AF could identify novel therapeutic targets. Additionally, leveraging advanced methodologies, such as multi-omics approaches, including genomics, proteomics, and metabolomics could uncover shared pathways contributing to both conditions. Subgroup analyses focusing on high-risk populations, such as those with obesity or obstructive sleep apnea, may help identify patients who could benefit most from targeted interventions [30]. These efforts will provide a clearer understanding of whether treating GERD can influence AF outcomes and potentially improve patient care.

First, only seven studies satisfied the inclusion criteria and were analyzed. Second, the lack of temporality in one cross-sectional study and one case-control study prevented us from determining the causal direction between GERD and AF. Third, notable variations existed among the studies regarding selection criteria, study design, statistical methods, and key findings. Lastly, subgroup analyses, such as examining factors like age, gender, and comorbidities that might influence AF risk in GERD, could not be conducted. Future large-scale prospective studies are needed to address these gaps. 

The generalizability of our findings should be considered within certain limitations. While our included studies spanned multiple countries and healthcare systems, they predominantly represented populations from developed nations with established healthcare infrastructure. Additionally, the heterogeneity in GERD severity definitions and varying comorbidity profiles across studies suggests that our results may be most applicable to patients with moderate GERD in well-resourced healthcare settings. Further research is needed to validate these findings in diverse healthcare contexts, particularly in low- and middle-income countries where GERD presentation and management may differ substantially.

## Conclusions

This meta-analysis demonstrates a significant association between GERD and an increased risk of AF, with a pooled relative risk of 1.27 (95% CI: 1.15-1.40). The relationship appears to be mediated through inflammatory pathways, where acid reflux-induced inflammation may extend from the esophagus to the left atrium. While Mendelian randomization studies suggest potential causality, further research is needed to clarify this relationship.

Given these findings, greater clinical awareness of AF risk in GERD patients, particularly those with additional risk factors such as older age and comorbidities, is warranted. Although current evidence is insufficient to recommend anti-GERD treatments for AF prevention, the potential for novel therapeutic strategies targeting both conditions should be explored. Future research should focus on large-scale prospective studies and clinical trials to better understand the association, refine risk stratification, and assess the efficacy of tailored treatment approaches.
